# Implementing treat-to-target urate-lowering therapy during hospitalizations for gout flares

**DOI:** 10.1093/rheumatology/kead574

**Published:** 2023-10-31

**Authors:** Mark D Russell, Louise Ameyaw-Kyeremeh, Flora Dell’Accio, Heather Lapham, Natalie Head, Christopher Stovin, Vishit Patel, Benjamin D Clarke, Deepak Nagra, Edward Alveyn, Maryam A Adas, Katie Bechman, María A de la Puente, Benjamin Ellis, Corrine Byrne, Rina Patel, Andrew I Rutherford, Fleur Cantle, Sam Norton, Edward Roddy, Joanna Hudson, Andrew P Cope, James B Galloway

**Affiliations:** Centre for Rheumatic Diseases, King’s College London, London, UK; Department of Rheumatology, King’s College Hospital NHS Foundation Trust, London, UK; Centre for Rheumatic Diseases, King’s College London, London, UK; Department of Rheumatology, King’s College Hospital NHS Foundation Trust, London, UK; Department of Rheumatology, King’s College Hospital NHS Foundation Trust, London, UK; Department of Rheumatology, King’s College Hospital NHS Foundation Trust, London, UK; Department of Rheumatology, King’s College Hospital NHS Foundation Trust, London, UK; Department of Rheumatology, King’s College Hospital NHS Foundation Trust, London, UK; Centre for Rheumatic Diseases, King’s College London, London, UK; Centre for Rheumatic Diseases, King’s College London, London, UK; Centre for Rheumatic Diseases, King’s College London, London, UK; Centre for Rheumatic Diseases, King’s College London, London, UK; Department of Psychology, Health Psychology Section, Institute of Psychiatry, Psychology, & Neuroscience, King's College London, London, UK; Department of Rheumatology, Imperial College Healthcare NHS Foundation Trust, London, UK; Pharmacy Department, King’s College Hospital NHS Foundation Trust, London, UK; Pharmacy Department, King’s College Hospital NHS Foundation Trust, London, UK; Department of Rheumatology, King’s College Hospital NHS Foundation Trust, London, UK; Department of Emergency Medicine, King’s College Hospital NHS Foundation Trust, London, UK; Centre for Rheumatic Diseases, King’s College London, London, UK; School of Medicine, Keele University, Keele, UK; Department of Psychology, Health Psychology Section, Institute of Psychiatry, Psychology, & Neuroscience, King's College London, London, UK; Centre for Rheumatic Diseases, King’s College London, London, UK; Centre for Rheumatic Diseases, King’s College London, London, UK

**Keywords:** gout, crystal arthritis, hospital, admissions, urate-lowering therapy, allopurinol

## Abstract

**Objectives:**

To evaluate a strategy designed to optimize care and increase uptake of urate-lowering therapy (ULT) during hospitalizations for gout flares.

**Methods:**

We conducted a prospective cohort study to evaluate a strategy that combined optimal in-hospital gout management with a nurse-led, follow-up appointment, followed by handover to primary care. Outcomes, including ULT initiation, urate target attainment and re-hospitalization rates, were compared between patients hospitalized for flares in the 12 months post-implementation and a retrospective cohort of hospitalized patients from 12 months pre-implementation.

**Results:**

One hundred and nineteen and 108 patients, respectively, were hospitalized for gout flares in the 12 months pre- and post-implementation. For patients with 6-month follow-up data available (*n* = 94 and *n* = 97, respectively), the proportion newly initiated on ULT increased from 49.2% pre-implementation to 92.3% post-implementation (age/sex-adjusted odds ratio [aOR] 11.5; 95% CI 4.36, 30.5; *P* < 0.001). After implementation, more patients achieved a serum urate ≤360 μmol/l within 6 months of discharge (10.6% pre-implementation *vs* 26.8% post-implementation; aOR 3.04; 95% CI 1.36, 6.78; *P* = 0.007). The proportion of patients re-hospitalized for flares was 14.9% pre-implementation *vs* 9.3% post-implementation (aOR 0.53; 95% CI 0.22, 1.32; *P* = 0.18).

**Conclusion:**

Over 90% of patients were initiated on ULT after implementing a strategy to optimize hospital gout care. Despite increased initiation of ULT during flares, recurrent hospitalizations were not more frequent following implementation. Significant relative improvements in urate target attainment were observed post-implementation; however, for the majority of hospitalized gout patients to achieve urate targets, closer primary–secondary care integration is still needed.

Rheumatology key messages

- After optimizing hospital gout care, more than 90% of patients were initiated on urate-lowering therapy.
- Significant relative improvements in urate target attainment were seen following a single, nurse-led, post-discharge appointment.
- However, for the majority of hospitalized gout patients to achieve target, closer primary–secondary care integration is still needed.

## Introduction

Hospitalizations for gout flares have increased markedly over the last 20 years, doubling in the USA, England and Canada [1–4]. These increases have occurred despite widespread availability of urate-lowering therapies (ULT), such as allopurinol and febuxostat. When titrated to achieve serum urate targets ≤360 μmol/l, ULT prevents flares, improves quality of life and leads to long-term reductions in hospitalizations [5–7]. International guidelines have been updated to encourage the uptake of treat-to-target ULT [8–11]. However, population-level data continue to show that ULT is initiated in only a minority of patients, while few patients achieve the urate targets necessary to prevent flares and hospitalizations [6, 12–14].

For avoidable gout admissions to be prevented, strategies are needed to optimize care and increase uptake of treat-to-target ULT in hospitalized patients. A recent systematic review found a paucity of high-quality studies in people hospitalized for gout [15]. Specifically, no prospective studies to date had evaluated strategies designed to encourage ULT uptake and prevent re-admissions in hospitalized patients [15]. We sought to address this knowledge gap.

In this study, we evaluated a strategy designed to optimize hospital gout care and increase uptake of ULT. Our strategy was modelled on a nurse-led intervention shown to be highly effective at optimizing gout management in primary care [5]. We adapted this strategy for implementation during hospitalizations for flares, and assessed outcomes including ULT initiation, serum urate target attainment and rates of re-hospitalization.

## Methods

### Study design and intervention

We performed a prospective cohort study at a large teaching hospital in South London, UK, which serves a population of over 1 million people. We evaluated outcomes after implementation of a strategy designed to optimize care for people hospitalized for gout flares, and compared these outcomes with a retrospective cohort of hospitalized patients from before implementation.

The intervention package consisted of two key components: (i) an in-hospital gout management pathway (Supplementary Data S1, available at *Rheumatology* online), based on British Society for Rheumatology (BSR), European Alliance of Associations for Rheumatology (EULAR), and American College of Rheumatology (ACR) gout management guidelines [8–10]; and (ii) a nurse-led telephone appointment performed 2 weeks after discharge.

The intervention was developed with extensive stakeholder input, following a systematic literature review [15], audit and process mapping of gout care at our hospital [16]. The management pathway was designed as a quick-reference guide on optimal gout care for use by frontline clinicians and rheumatologists. This included recommendations on: diagnostic tests (including serum urate levels and joint aspiration); rheumatologist input; flare treatments (NSAIDs, colchicine and/or corticosteroids, where appropriate); offering ULT (allopurinol first-line) to all patients unless contraindicated; initiating ULT during the acute flare; considering prophylaxis against flares during ULT initiation and titration; admission-avoidance strategies (e.g. ambulatory care units); disease education; and post-discharge advice (including treat-to-target ULT optimization, as recommended in the BSR gout management guideline [8]).

A nurse-led telephone clinic was established to provide patients with a single follow-up appointment within 2 weeks of discharge. This clinic was delivered on a weekly basis by a specialist rheumatology nurse, trained in gout management, with appointments lasting ∼30 min per patient. Objectives were to review symptoms, provide disease education, discuss flare management strategies, and provide advice to patients and their primary care team on ULT dose optimization using a treat-to-target strategy [8]. After this appointment, care was handed over to the patient’s primary care team via a clinical letter. For patients with severe gout and/or recurrent admissions, additional rheumatology outpatient follow-up could be considered.

To maximize uptake of the intervention, a multi-pronged implementation strategy was developed with implementation experts. This incorporated strategies from the Expert Recommendations for Implementing Change (ERIC) guidance [17], including digital order sets, study champions, education sessions, advertising, executive approval, quality monitoring and clinician feedback (Supplementary Data S2, available at *Rheumatology* online).

### Study period

The study period was from 30 October 2020 to 29 April 2023. The intervention was launched on 30 October 2021. Data were collected prospectively on all patients hospitalized with gout flares in the 12-month period after intervention launch (30 October 2021–29 October 2022). Post-implementation outcomes were compared with outcomes for patients hospitalized with gout in the 12-month period prior to launch (30 October 2020–29 October 2021). All patients with linked primary care data were followed up for 6 months after discharge to review post-discharge outcomes (detailed below).

### Case definitions

All emergency department (ED) attendances and admission episodes for gout flares (collectively referred to as hospitalizations) in the pre- and post-implementation periods were included. Both primary and secondary admission diagnoses of gout (e.g. flares occurring during hospitalizations for other reasons) were eligible, assuming gout was deemed the likely cause of the acute joint symptoms by the primary clinical team. Although recommended in our pathway, confirmatory joint aspiration and/or rheumatology input were not mandated, as reflects local clinical practice. Patients managed solely in an urgent care centre (primarily staffed by general practitioners rather than ED clinicians) were excluded, as the urgent care centre facility at our hospital was transferred to another institution prior to intervention launch.

### Data sources

All data used in these analyses were routinely captured during clinical care. In-hospital data were extracted from electronic patient records. Post-discharge data were extracted from local care records, containing primary and secondary care data for patients with linked NHS identifiers who had not opted out of this service [18]. All data were manually validated by a rheumatologist, and pseudonymized for the purposes of analysis. Outcomes were selected a priori with stakeholder input.

### Baseline characteristics

Baseline data were collected as follows: age; sex; admission type (ED attendance-only *vs* hospital admission); day/time of presentation (defined as out-of-hours if occurring between 9 p.m. and 9 a.m. or on a Saturday/Sunday); pre-existing gout diagnosis; pre-existing prescription for ULT (allopurinol, febuxostat, benzbromarone, sulfinpyrazone or probenecid); and baseline blood tests, if performed during the presentation (serum urate, CRP, white cell count, neutrophil count, lymphocyte count and serum creatinine).

### Outcomes during hospitalization

Data were captured to ascertain whether the following outcomes occurred during the hospitalization episode: rheumatology input sought; serum urate level performed; joint aspiration performed; flare treatment(s) prescribed (NSAID, colchicine and/or oral, intramuscular, intravenous or intra-articular corticosteroids); disease education provided to patients; ULT initiated (if patient not already receiving ULT) or up-titrated (if patient already receiving ULT at a sub-optimal dose); prophylaxis prescribed (low-dose colchicine, NSAIDs or corticosteroids); gout-specific recommendations and/or follow-up on discharge.

### Outcomes after hospitalization

For patients with available follow-up data, we ascertained whether the following outcomes occurred within 6 months of discharge: ULT initiation and/or up-titration; prescription of prophylaxis against flares during ULT initiation/titration; number of serum urate levels performed; attainment of serum urate targets ≤360 μmol/l and/or ≤300 μmol/l; follow-up in the gout telephone clinic and/or rheumatology outpatient clinic; re-attendance at ED and/or re-admission with a subsequent gout flare (occurring >7 days after discharge from the initial presentation).

### Statistical methods

Baseline characteristics were tabulated, and between-cohort differences estimated using the chi-square test for categorical variables and an independent Student’s *t*-test for continuous variables. Logistic regression, with adjustment for age and sex, was used to estimate differences in categorical outcomes between pre- and post-implementation cohorts, expressed as adjusted odds ratios (aOR) with 95% CI. Linear regression, with adjustment for age and sex, was used to estimate differences in continuous outcomes, expressed as adjusted β-coefficients (aβ) with 95% CI. Kaplan–Meier survival curves were presented for repeat hospitalizations. Univariable logistic regression was performed to explore a differential impact of the intervention on patient subgroups, categorized by age, sex, admission type, time of presentation, whether the gout diagnosis was pre-existing, or whether rheumatology input was sought during hospitalization. Stata v17 (StataCorp, College Station, TX, USA) was used for all analyses. No adjustment was performed for multiple hypothesis testing, as this was an exploratory study.

### Study approval and ethics

Approval to undertake this study under the remit of service evaluation was obtained from King’s College Hospital NHS Foundation Trust. No further ethical approval or written informed patient consent was required, as per UK Health Research Authority guidance.

### Patient and public involvement

Patients have been closely involved in all stages of this project. Patient feedback was instrumental in conceptualizing this project, and in designing the intervention. In particular, patients emphasized the importance of a holistic, multi-faceted intervention and implementation strategy, recognizing that a single intervention was unlikely to address the multiple barriers to optimal hospital gout care. Patients will be closely involved in disseminating the findings of this study, and in developing follow-on projects.

## Results

### Baseline characteristics

**Figure 1.**
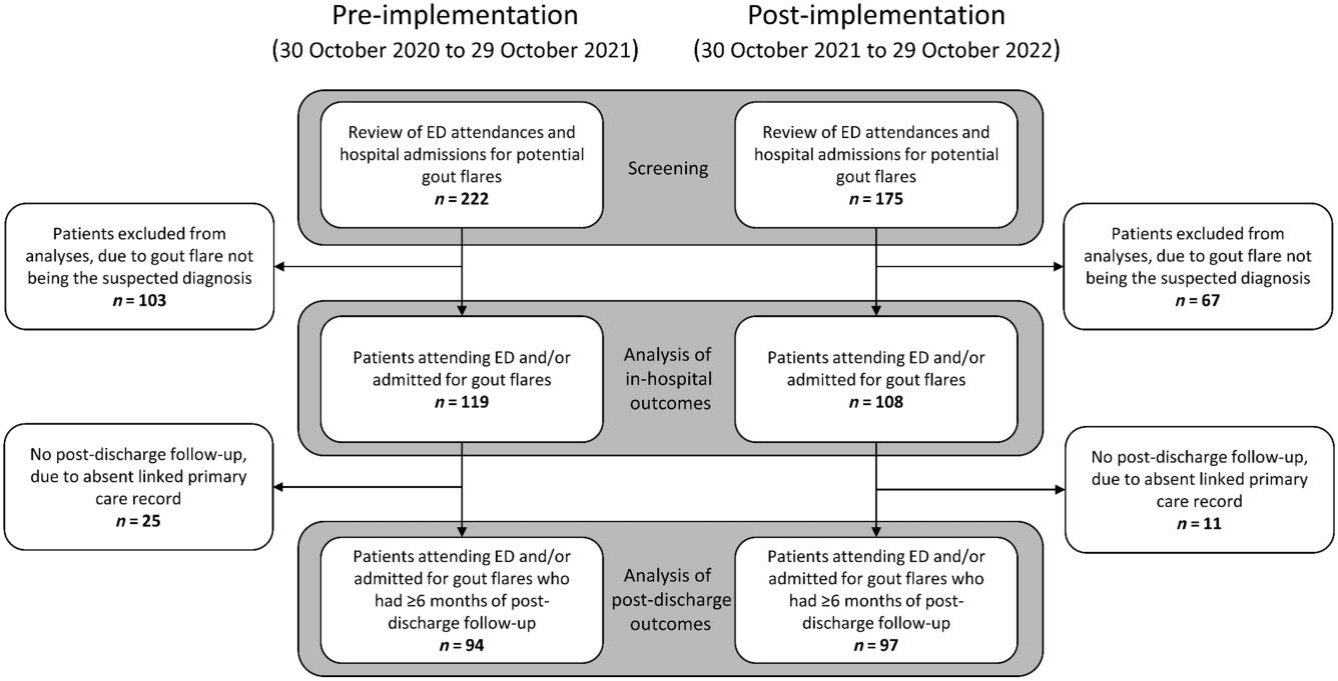
Study flowchart depicting the pre- and post-implementation study cohorts. ED: emergency department

In the 12 months prior to implementation of the intervention, 119 people attended ED with gout flares, of whom 63 (52.9%) required admission to hospital. In the 12 months after implementation, 108 attended ED with gout flares, of whom 53 (49.1%) required admission. A study flowchart is shown in Fig. 1.

Baseline characteristics are shown in Table 1. Pre- and post-implementation cohorts had similar mean ages (62 *vs* 64 years, respectively). There were proportionally more female patients in the pre- than post-implementation cohort (26.9% *vs* 15.7%). The proportion of patients who had pre-existing gout diagnoses was similar in the pre- and post-implementation cohorts (66.4% *vs* 67.6%); 41.8% and 27.4% of known gout patients in the pre- and post-implementation cohorts, respectively, were receiving ULT prior to hospitalization. Mean serum urate levels at baseline were comparable (485 *vs* 487 μmol/l).

**Table 1.** Baseline characteristics of the pre- and post-implementation cohorts

Characteristic	Pre-implementation (*n* = 119)	Post-implementation (*n* = 108)	*P*-value
Age, mean (s.d.), years	62 (16)	64 (16)	0.31
Sex, *n* (%)			
Female	32 (26.9)	17 (15.7)	0.041
Male	87 (73.1)	91 (84.3)	
Admission type, *n* (%)			
Discharged from ED	56 (47.1)	55 (50.9)	0.56
Admitted to hospital	63 (52.9)	53 (49.1)	
Presented out-of-hours, *n* (%)	59 (49.6)	43 (39.8)	0.14
Pre-existing gout diagnosis, *n* (%)	79 (66.4)	73 (67.6)	0.85
Receiving ULT prior to hospitalization, *n* (%)	33 (41.8)	20 (27.4)	0.063
Not on ULT prior to hospitalization, *n* (%)	46 (58.2)	53 (72.6)	
Serum urate at baseline, mean (s.d.), μmol/l	485 (185)	487 (125)	0.94
CRP at baseline, mean (s.d.), mg/l	89 (84)	76 (79)	0.28
White cell count at baseline, mean (s.d.), ×10^9^/l	9.3 (3.5)	9.5 (4.6)	0.76
Neutrophil count at baseline, mean (s.d.), ×10^9^/l	6.6 (3.1)	6.5 (3.9)	0.79
Lymphocyte count at baseline, mean (s.d.), ×10^9^/l	1.6 (0.8)	1.8 (0.9)	0.14
Creatinine at baseline, mean (s.d.), μmol/l	165 (161)	142 (116)	0.24

Blood test results represent the first tests performed during the hospitalization. Inferential statistics for between-cohort differences were obtained from independent *t*-tests for continuous variables and chi-square tests for categorical variables. ULT: urate-lowering therapy.

### Outcomes during hospitalizations

In-hospital outcomes were compared before and after implementation (Table 2). Following implementation, specialist rheumatology input was obtained more frequently in hospital (54.6% pre-implementation *vs* 75.9% post-implementation; aOR 2.48; 95% CI 1.37, 4.52; *P* = 0.003); serum urate levels were performed in more patients (66.4% *vs* 92.6%; aOR 6.32; 95% CI 2.75, 14.5; *P* < 0.001); and joint aspiration was performed more frequently (19.3% *vs* 47.2%; aOR 3.44; 95% CI 1.88, 6.27; *P* < 0.001).

**Table 2.** Outcomes during hospitalizations for gout flares, comparing the pre- and post-implementation cohorts

Outcome	**Pre-implementation, *n* (%) (*n* = 119)**	Post-implementation, *n* (%) (*n* = 108)	Odds ratio (95% CI)	*P*-value
Rheumatology input during hospitalization	65 (54.6)	82 (75.9)	2.48 (1.37, 4.52)	0.003
Serum urate level performed	79 (66.4)	100 (92.6)	6.32 (2.75, 14.5)	<0.001
Joint aspiration performed	23 (19.3)	51 (47.2)	3.44 (1.88, 6.27)	<0.001
Flare treatment prescribed	111 (93.3)	106 (98.1)	4.46 (0.91, 21.8)	0.065
NSAIDs	38 (31.9)	34 (31.5)	1.18 (0.61, 2.28)	0.63
Colchicine	74 (62.2)	86 (79.6)	2.30 (1.23, 4.31)	0.009
Corticosteroids	25 (21.0)	40 (37.0)	2.20 (1.21, 4.02)	0.010
Multiple flare treatments prescribed	21 (17.6)	49 (45.4)	4.10 (2.20, 7.67)	<0.001
Intra-articular steroid injection	2 (1.7)	9 (8.3)	5.53 (1.15, 26.7)	0.033
Disease education documented prior to discharge	27 (22.7)	24 (22.2)	1.00 (0.53, 1.90)	0.99
ULT initiated and/or titrated during hospitalization	21 (17.6)	67 (62.0)	7.69 (4.12, 14.4)	<0.001
Gout recommendations documented on discharge	70 (58.8)	93 (86.1)	4.33 (2.21, 8.48)	<0.001
Recommendation to initiate and/or titrate ULT after discharge	18 (15.1)	42 (38.9)	3.26 (1.71, 6.19)	<0.001
Recommendation for prophylaxis while titrating ULT	23 (19.3)	13 (12.0)	0.54 (0.26, 1.15)	0.11
Recommendation for target serum urate level	13 (10.9)	28 (25.9)	2.56 (1.23, 5.36)	0.012
Recommendation for primary care follow-up	53 (44.5)	43 (39.8)	0.82 (0.48, 1.41)	0.47
Recommendation for rheumatology/gout clinic follow-up	11 (9.2)	73 (67.6)	19.8 (9.34, 42.0)	<0.001

Odds ratios from logistic regression models are shown, with adjustment for age and sex. ULT: urate-lowering therapy.

Of pre- and post-implementation cohorts, 93.3% and 98.1%, respectively, received a guideline-recommended flare treatment. After implementation, more patients were prescribed colchicine (62.2% *vs* 79.6%; aOR 2.30; 95% CI 1.23, 4.31; *P* = 0.009), corticosteroids (21.0% *vs* 37.0%; aOR 2.20; 95% CI 1.21, 4.02; *P* = 0.010) or multiple flare treatments (17.6% *vs* 45.4%; aOR 4.10; 95% CI 2.20, 7.67; *P* < 0.001). Use of intra-articular corticosteroids increased modestly from a low baseline (1.7% *vs* 8.3%; aOR 5.53; 95% CI 1.15, 26.7; *P* = 0.033). There was no significant difference in the use of NSAIDs (31.9% *vs* 31.5%; aOR 1.18; 95% CI 0.61, 2.28; *P* = 0.63).

The proportion of patients initiated and/or up-titrated on ULT prior to discharge increased markedly following implementation, from 17.6% to 62.0% (aOR 7.69; 95% CI 4.12, 14.4; *P* < 0.001). After implementation, more patients were provided with gout-specific management recommendations on discharge (58.8% *vs* 86.1%; aOR 4.33; 95% CI 2.21, 8.48; *P* < 0.001). Documented evidence of disease education provision prior to discharge was low in both cohorts (22.7% *vs* 22.2%; aOR 1.00; 95% CI 0.53, 1.90; *P* = 0.99).

### Outcomes after hospitalizations

Of the patients in the pre- and post-implementation cohorts, 94/119 (79.0%) and 97/108 (89.8%), respectively, had primary care follow-up data available to facilitate analyses of post-discharge outcomes (Table 3). By 6 months post-discharge, 91/97 (93.8%) of the post-implementation cohort were prescribed ULT, compared with 61/94 (64.9%) pre-implementation (aOR 7.68; 95% CI 3.02, 19.6; *P* < 0.001). When restricted to patients not receiving ULT prior to hospitalization, the proportion of patients who newly initiated ULT in hospital or within 6 months of discharge increased markedly after implementation, from 49.2% to 92.3% (aOR 11.5; 95% CI 4.36, 30.5; *P* < 0.001). Of all patients receiving ULT by 6 months, 57/61 (93.4%) and 90/91 (98.9%) of the pre- and post-implementation cohorts, respectively, were prescribed allopurinol. There was no significant difference in prophylaxis use between patients newly initiating ULT in the pre- *vs* post-implementation periods (25.0% *vs* 29.2%; aOR 1.12; 95% CI 0.42, 2.98; *P* = 0.81).

**Table 3.** Outcomes in the 6-month period after hospitalizations for gout flares, comparing the pre- and post-implementation cohorts

Outcome	**Pre-implementation, *n* (%) (*n* = 94)**	Post-implementation, *n* (%) (*n* = 97)	Odds ratio (95% CI)	*P*-value
Receiving ULT by 6 months	61 (64.9)	91 (93.8)	7.68 (3.02, 19.6)	<0.001
ULT initiated in hospital or within 6 months of discharge				
Yes	32 (49.2)	72 (92.3)	11.5 (4.36, 30.5)	<0.001
No	33 (50.8)	6 (7.7)		
Receiving ULT pre-admission	29	19		
Prophylaxis prescribed while initiating ULT				
Yes	8 (25.0)	21 (29.2)	1.12 (0.42, 2.98)	0.81
No	24 (75.0)	51 (70.8)		
Not newly initiated on ULT	62	25		
Serum urate performed at least once within 6 months	30 (31.9)	56 (57.7)	2.88 (1.58, 5.25)	0.001
Serum urate ≤360 μmol/l within 6 months	10 (10.6)	26 (26.8)	3.04 (1.36, 6.78)	0.007
Serum urate ≤300 μmol/l within 6 months	5 (5.3)	13 (13.4)	2.65 (0.89, 7.84)	0.079
Rheumatology outpatient clinic within 6 months	8 (8.5)	16 (16.5)	2.08 (0.82, 5.28)	0.12
Gout telephone clinic within 6 months	N/A	79 (81.4)	—	—
Re-presented to hospital within 6 months	14 (14.9)	9 (9.3)	0.53 (0.22, 1.32)	0.18

Odds ratios from logistic regression models are shown, with adjustment for age and sex. A gout telephone clinic was established as part of the intervention package, and therefore was not available to patients in the pre-implementation cohort. N/A: not available; ULT: urate-lowering therapy.

Following implementation, more patients achieved a serum urate ≤360 μmol/l within 6 months of discharge: 10/94 (10.6%) pre-implementation *vs* 26/97 (26.8%) post-implementation (aOR 3.04; 95% CI 1.36, 6.78; *P* = 0.007). There was no significant difference in the proportion of patients achieving a serum urate ≤300 μmol/l within 6 months (5.3% pre-implementation *vs* 13.4% post-implementation; aOR 2.65; 95% CI 0.89, 7.84; *P* = 0.079). Mean reductions in serum urate at 6 months, relative to baseline, were 29.7 μmol/l pre-implementation *vs* 96.8 μmol/l post-implementation (aβ −64.3; 95% CI −128.0, −0.64; *P* = 0.048). The mean number of serum urate levels performed within 6 months increased from 0.5 tests pre-implementation to 1.1 tests post-implementation (aβ 0.55; 95% CI 0.14, 0.96; *P* = 0.009).

Of the post-implementation cohort, 79/97 (81.4%) were reviewed in the nurse-led gout telephone clinic (median time to review: 12 days), while 16/97 (16.5%) patients received rheumatology outpatient follow-up within 6 months. Prior to implementation, 8/94 (8.5%) patients received rheumatology outpatient follow-up within 6 months of discharge.

**Figure 2.**
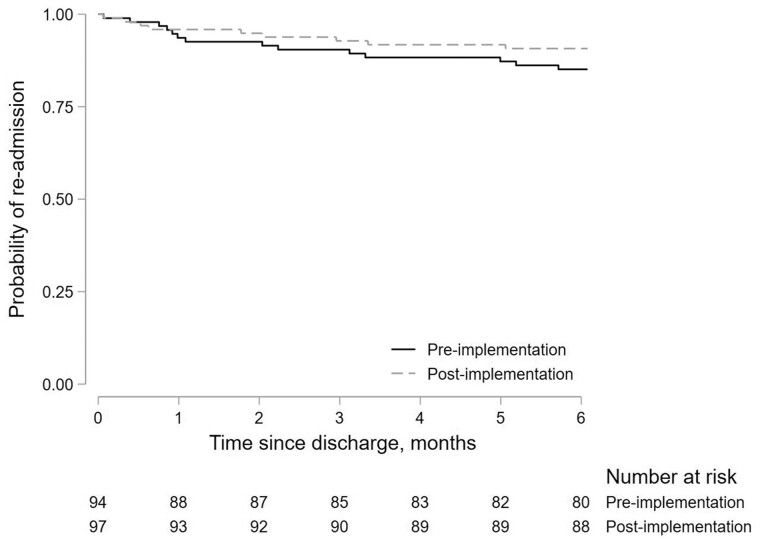
Survival curve showing the probability of re-attendance at ED and/or re-admission to hospital for gout flares following discharge. Pre-implementation (solid line) and post-implementation (dashed line) cohorts are shown. The number of patients at risk at each time point is shown in a risk table

The number of patients who re-attended ED and/or were re-admitted for gout flares within 6 months of discharge was 14/94 (14.9%) pre-implementation *vs* 9/97 (9.3%) post-implementation (aOR 0.53; 95% CI 0.22, 1.32; *P* = 0.18). Survival curves for repeat hospitalizations are shown in Fig. 2.

Further analyses were performed to explore the impact of the intervention on different subgroups of patients (Supplementary Figs S1–S4, available at *Rheumatology* online)*.* Odds of ULT initiation were significantly higher in the post-implementation than pre-implementation cohort, irrespective of age, sex, admission type, time of presentation, whether the gout diagnosis was pre-existing or whether rheumatology input was sought during hospitalization (Supplementary Fig. S1, available at *Rheumatology* online).

## Discussion

Following implementation of a strategy designed to optimize care during gout hospitalizations, >90% of ULT-naïve patients were initiated on ULT—nearly double the pre-implementation baseline. Many other aspects of care improved, including urate target attainment and post-discharge follow-up. The initiation of ULT during flares did not increase recurrent hospitalizations, supporting the use of ULT in this setting.

Our intervention was modelled on one shown to be highly effective in a primary care setting. In a large randomized controlled trial in the UK, nurse-delivered patient education and treat-to-target ULT resulted in 95% of patients achieving serum urate targets within 1 year, compared with 26% with usual care [5]. Flare frequency, tophi and quality of life all improved, and the intervention was shown to be cost-effective. We adapted this intervention for implementation in a hospital setting. As well as optimizing care during patients’ hospital stays, we established a nurse-led, post-discharge clinic to facilitate disease education and provide advice on ULT optimization. This appointment was delivered as a single telephone appointment, recognizing that in-person appointments can be challenging for patients to attend after hospitalizations for flares. Care was then handed over to patients’ primary care teams for ongoing management.

Following implementation of this strategy, many aspects of hospital gout care improved: joint aspirations increased; serum urate levels were performed more frequently; use of guideline-recommended flare treatments increased (particularly combination therapy); and gout-specific follow-up was provided to more patients. Rheumatologist input also increased; specialist support for frontline clinicians was felt to be an important facilitator of optimal gout care during our stakeholder consultations [16], supported by previous analyses demonstrating that rheumatology input associates with improvements in care for hospitalized gout patients [19–22].

The biggest change observed following implementation of our strategy was increased initiation of ULT. By 6 months post-discharge, 94% of patients had been prescribed ULT. This is comparable to ULT initiation rates in the Nottingham primary care-based study [5], and substantially better than the 61% of patients who were receiving ULT within 12 months of hospitalization in a recent UK-wide analysis [6]. In particular, there was a 3-fold increase in the proportion of patients initiating and/or up-titrating ULT prior to discharge. There has been extensive debate around the relative benefits and harms of early ULT initiation (*vs* deferred initiation of ULT after flare resolution), with international guidelines varying widely in this regard [8, 10, 11]. We advocated for early ULT initiation for several reasons. First, hospitalizations provide unique opportunities for clinicians to optimize care for people with long-term conditions, such as gout. Second, accumulating evidence suggests that upfront initiation of ULT does not prolong or worsen intercurrent flares, provided it is initiated alongside flare treatment [23–26]. Third, earlier initiation of ULT leads to more timely reductions in serum urate levels [23–26]. Finally, this approach can help to mitigate a breakdown in communication between secondary care and primary care, whereby post-discharge recommendations to initiate ULT are not acted upon [19].

Despite the marked increase in ULT initiation during flares, we did not observe an increase in hospitalizations for recurrent flares after implementing this strategy. These real-world data support those obtained from trial settings [23–26]. Although not statistically significant, proportionately fewer re-hospitalizations occurred after implementation, relative to before (37.6% relative reduction; 5.6% absolute reduction), suggesting a potential for benefit with this approach. One contributory factor might have been the post-discharge follow-up appointment, which gave patients an opportunity to have any ongoing symptoms reviewed. Advice on flare management was provided within this appointment, empowering patients to self-manage flares. With longer follow-up, there is the potential for more admissions to be prevented with this strategy: observational data show that ULT associates with a significantly reduced risk of recurrent hospitalizations from 12 months after initiation, particularly when urate targets are attained [6]. Future work will help to determine whether primary care workload is also reduced following implementation of better hospital gout care.

Prior to implementation, only 10% of patients achieved a serum urate ≤360 μmol/l within 6 months of discharge. After implementation, urate target attainment more than doubled, to 26.8%. Despite this relative improvement, absolute levels of urate target attainment remained far below those seen in the Nottingham primary care trial (95% attainment by 12 months) [5]. Similarly, target attainment was below that reported in the BSR National Audit of outpatient gout management by UK rheumatologists (45% attainment by 12 months) [27], and only modestly better than what was reported in a UK-wide analysis of post-discharge gout care (1184/7040 [16.8%] patients attaining urate ≤360 μmol/l within 12 months of hospitalization [6]). There are several possible reasons for this. Follow-up in our study was relatively short at only 6 months. Patients in our study were all hospitalized for gout, and therefore are likely to represent a more severe cohort. Perhaps most importantly, in the Nottingham study there were an average of 17 study visits per participant over a 24-month period. In contrast, our gout follow-up clinic was delivered as a single telephone appointment, followed by handover of care to patients’ primary care teams. Indeed, target attainment in our study was comparable to that seen in the usual care group of the Nottingham trial (26.8% *vs* 26.2%, respectively). Thus, our findings strongly suggest that our intervention, while effective at facilitating ULT initiation, is insufficient for the majority of people hospitalized for gout flares to achieve target urate levels.

There are several ways in which our intervention could be altered to promote urate target attainment. Rheumatologists could take greater ownership of hospitalized gout patients by providing outpatient follow-up until urate targets are achieved. Alternatively, training could be provided for healthcare professionals in primary care (e.g. nurses and/or pharmacists) to deliver optimal treat-to-target ULT, which was shown to be highly effective in the Nottingham study [5], NOR-Gout study [7] and many other studies [28, 29]. Strategies could be modelled on other integrated care services, which proactively identify patients in hospital before transferring them to primary care-based pathways with secondary care support, such as fracture liaison services [30]. Future strategies should also encourage adherence to ULT, which although not assessed in our study, is often sub-optimal and may have contributed to our finding of infrequent urate target attainment despite high levels of ULT initiation [31]; this could incorporate patient education programmes, self-management tools and point-of-care urate testing [30].

A key strength of our study was the close involvement of stakeholders, patients and methodologists when developing our intervention and implementation strategy. Our intervention was based on best practice care from national and international gout management guidelines [8–11], and was modelled on the highly successful intervention used in the Nottingham primary care trial [5]. We adopted a multi-faceted implementation strategy to maximize intervention uptake. This incorporated several implementation strategies recommended in the ERIC guidance [17], including digital enablers, study champions, education sessions and clinician feedback. Adopting a multi-faceted strategy is particularly important when implementing complex interventions in healthcare settings. For example, an implementation strategy involving only educational sessions for clinicians in emergency departments may not succeed, given the challenges of reaching all frontline staff.

Our study also had limitations. Follow-up was only 6 months, which may have been too short to ascertain differences in post-discharge outcomes such as urate target attainment and recurrent hospitalizations. While the numerical reductions in re-hospitalizations we observed following implementation of our strategy might have been clinically meaningful, our study was underpowered to detect significant differences in these relatively rare events. Use of prophylaxis against flares whilst initiating/titrating ULT remained infrequent despite the intervention, particularly when compared with national data [27], and future studies should encourage the use of prophylaxis, given the benefits on flare reduction [8]. Data on comorbidities and other clinical outcomes (e.g. flare frequency or tophus burden) were unavailable. We included all hospitalizations where gout was deemed the likely diagnosis; crystal analysis and rheumatologist input were recommended, but not mandated. These pragmatic inclusion criteria reflect real-world clinical practice, although there remains a potential for diagnostic misclassification. Additionally, we utilized a retrospective comparator, rather than a prospective comparator. This was an a priori decision, to reflect resource availability and the service evaluation remit of this project; however, it is possible that some of the changes observed may represent changes in practice over time, rather than a direct result of the intervention (e.g. changes in service delivery during the COVID-19 pandemic). Our findings should therefore be seen as exploratory, rather than definitive. Finally, as our analyses were conducted at a single UK centre, the findings cannot be assumed to be generalizable to other healthcare settings.

In conclusion, after implementing a strategy designed to optimize care for people hospitalized with gout flares, >90% of patients were initiated on ULT. In the context of a single, nurse-led follow-up appointment, relative improvements in urate target attainment were observed; however, for the majority of hospitalized gout patients to achieve target urate levels, better in-hospital gout care needs to be accompanied by strategies that embed and support optimization of ULT in primary care.

## Supplementary Material

kead574_Supplementary_Data

## Data Availability

The data underlying this article cannot be shared publicly to ensure the privacy of individuals that participated in the study.
