# Supplementary material for: Implementing treat-to-target urate-lowering therapy during hospitalizations for gout flares
Source: Rheumatology (Oxford). 2023 Oct 31;63(8):2222–9. doi: 10.1093/rheumatology/kead574 (PMC11292051; doi:10.1093/rheumatology/kead574)
Supplement: kead574_Supplementary_Data [file kead574_supplementary_data.docx]

**Supplementary Data S1:** Pathway for the management of patients attending emergency department (ED) and/or admitted for gout flares.


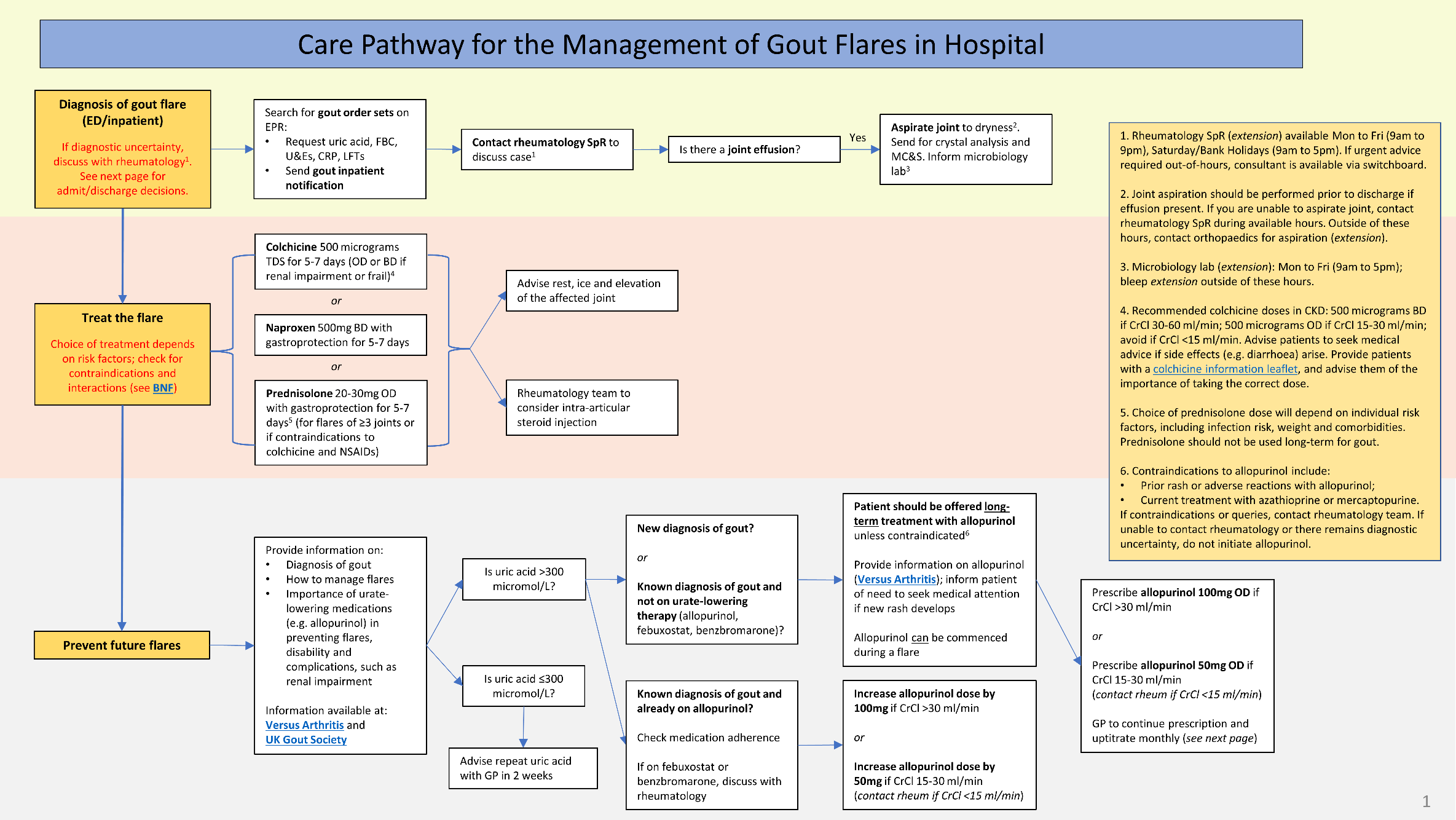


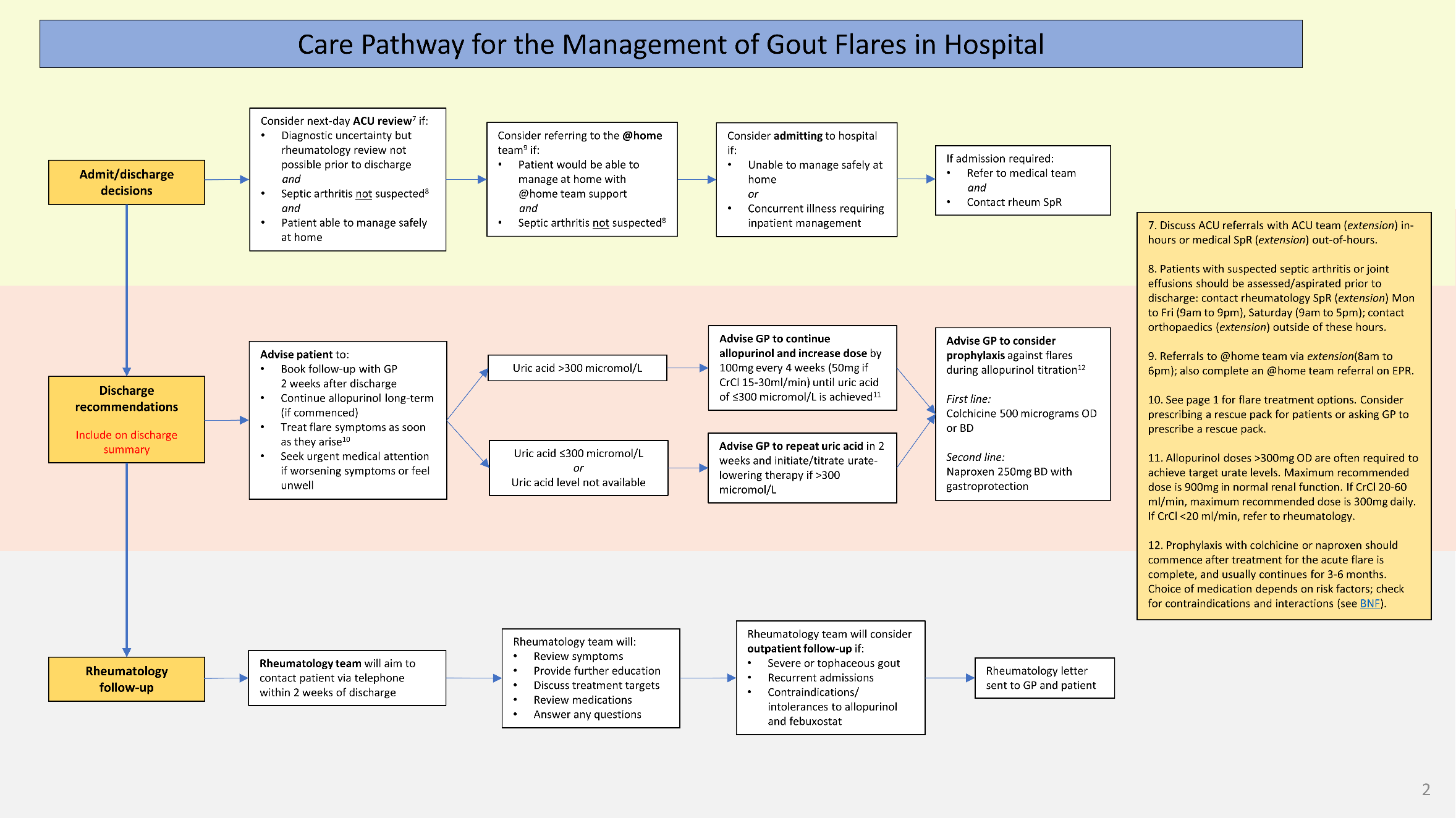


EPR: electronic patient record; SpR: specialty registrar doctor; MC&S: microscopy, culture and sensitivity; OD: once daily; BD: twice daily; TDS: three times daily; BNF: British National Formulary; GP: general/family practitioner; CrCl; creatinine clearance; ACU: ambulatory care unit; @home team: at-home treatment outreach team.

**Supplementary Data S2:** Implementation strategy.

Strategies were selected from the Expert Recommendations for Implementing Change guidance, and tailored to the specific hospital environment. These included:

- **Patient involvement** – patients were closely involved in all stages of the project, from conceptualisation to intervention design and dissemination of the findings. Feedback was obtained from patients on the care they received under the pathway.
- **Digital enablers** – order sets were created for relevant investigations (e.g. serum urate; synovial fluid crystal analysis) and medications (e.g. flare treatment options; urate-lowering therapy) within the hospital’s electronic health record system. Additionally, an eNotification system was created, whereby clinicians could complete an electronic order form, which would notify the rheumatology team when a patient had been hospitalised for gout.
- **Study champions** – clinicians from several specialties (e.g. emergency medicine; rheumatology) were provided with the opportunity to take part in developing the intervention, and in supporting its implementation within their department.
- **Implementation experts** – advice was sought from experts on the implementation of complex healthcare interventions when designed the intervention and implementation strategy.
- **Educational sessions** – in-person and virtual training sessions were delivered for clinicians from several departments (including emergency medicine, acute medicine, general medicine and rheumatology), to familiarise them with the pathway, provide guidance on optimal gout management, and answer questions. These sessions were staggered over time, to enable as many clinicians as possible to attend.
- **Executive approval** – hospital executives were involved in all stages of the project, including intervention development and design. Formal approval and hospital sign-off were obtained for the pathway, prior to its implementation, such that it became an official hospital care pathway.
- **Advertising** – information on the pathway, training sessions and order sets were uploaded to the hospital intranet, and circulated to all staff via email. Additionally, tailored emails were sent to individual departments, notifying them of the pathway and its implementation.
- **Clinical supervision** – rheumatology specialists with expertise in gout management were available to support clinicians in optimising gout care for patients during their admission.
- **Quality monitoring and clinician feedback** – outcomes were monitored prospectively throughout the intervention period. Opportunities for clinician feedback were provided, and any questions on the pathway were answered; however, no changes were made to the pathway itself during the study period.


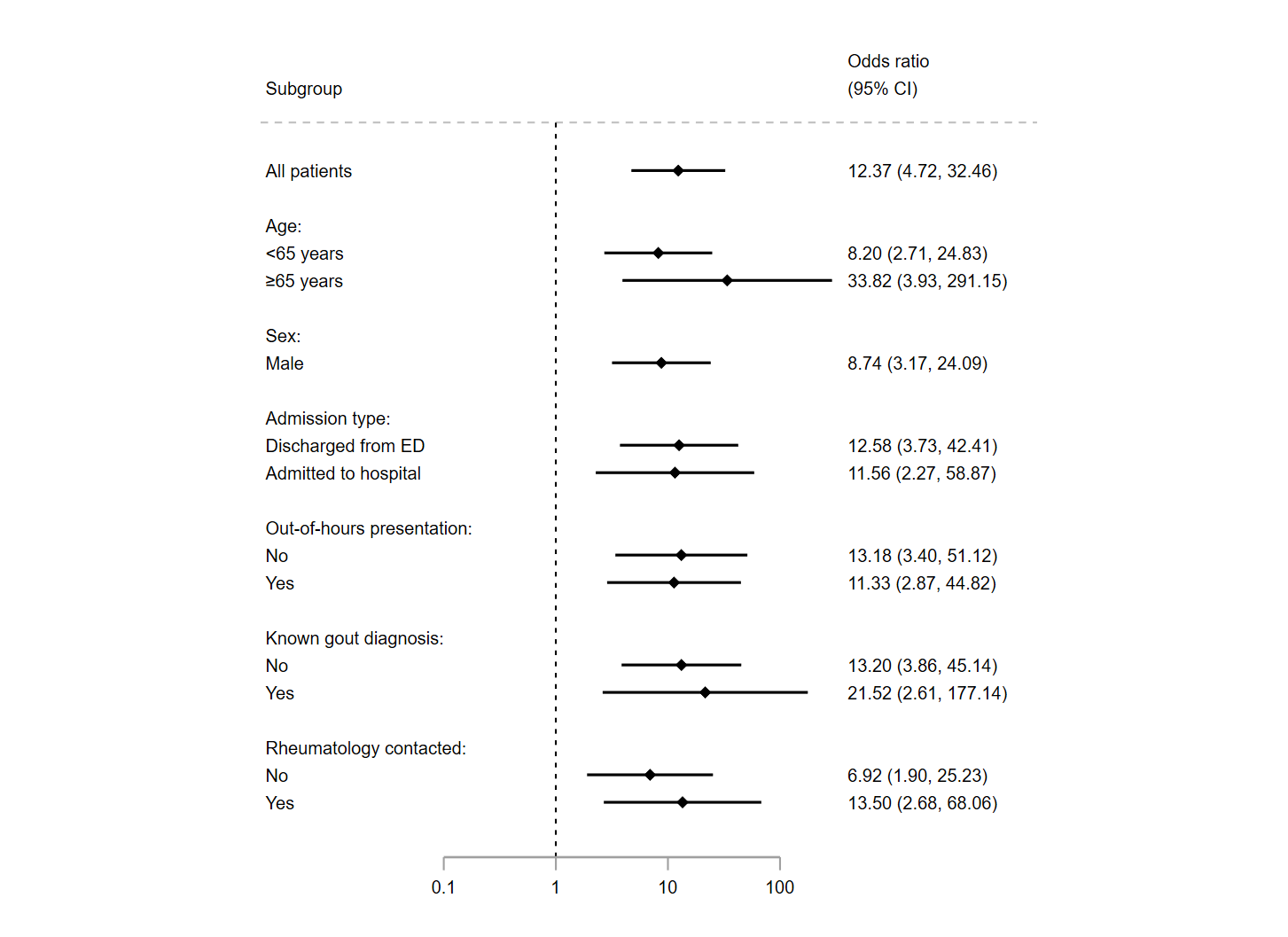
**Supplementary Figure S1.** Odds of being initiated on ULT during hospitalisation or within 6 months of discharge in the post-implementation cohort relative to the pre-implementation cohort, comparing different subgroups of patients.

Outputs shown are from univariable logistic regression models. Female sex is omitted, as all female patients were receiving ULT by 6 months post-discharge.


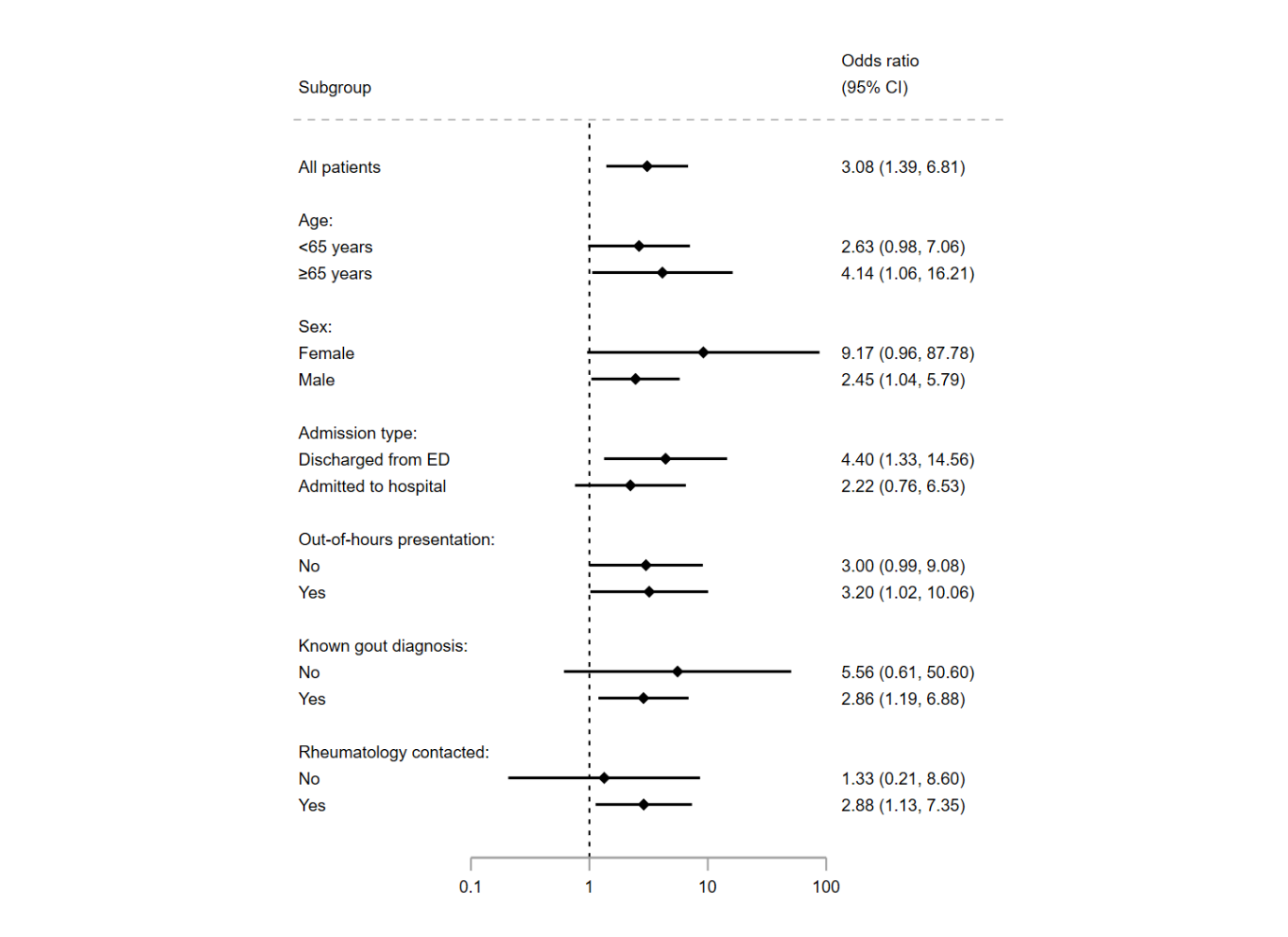
**Supplementary Figure S2.** Odds of achieving a serum urate ≤360 micromol/L within 6 months of discharge in the post-implementation cohort relative to the pre-implementation cohort, comparing different subgroups of patients.

Outputs shown are from univariable logistic regression models.


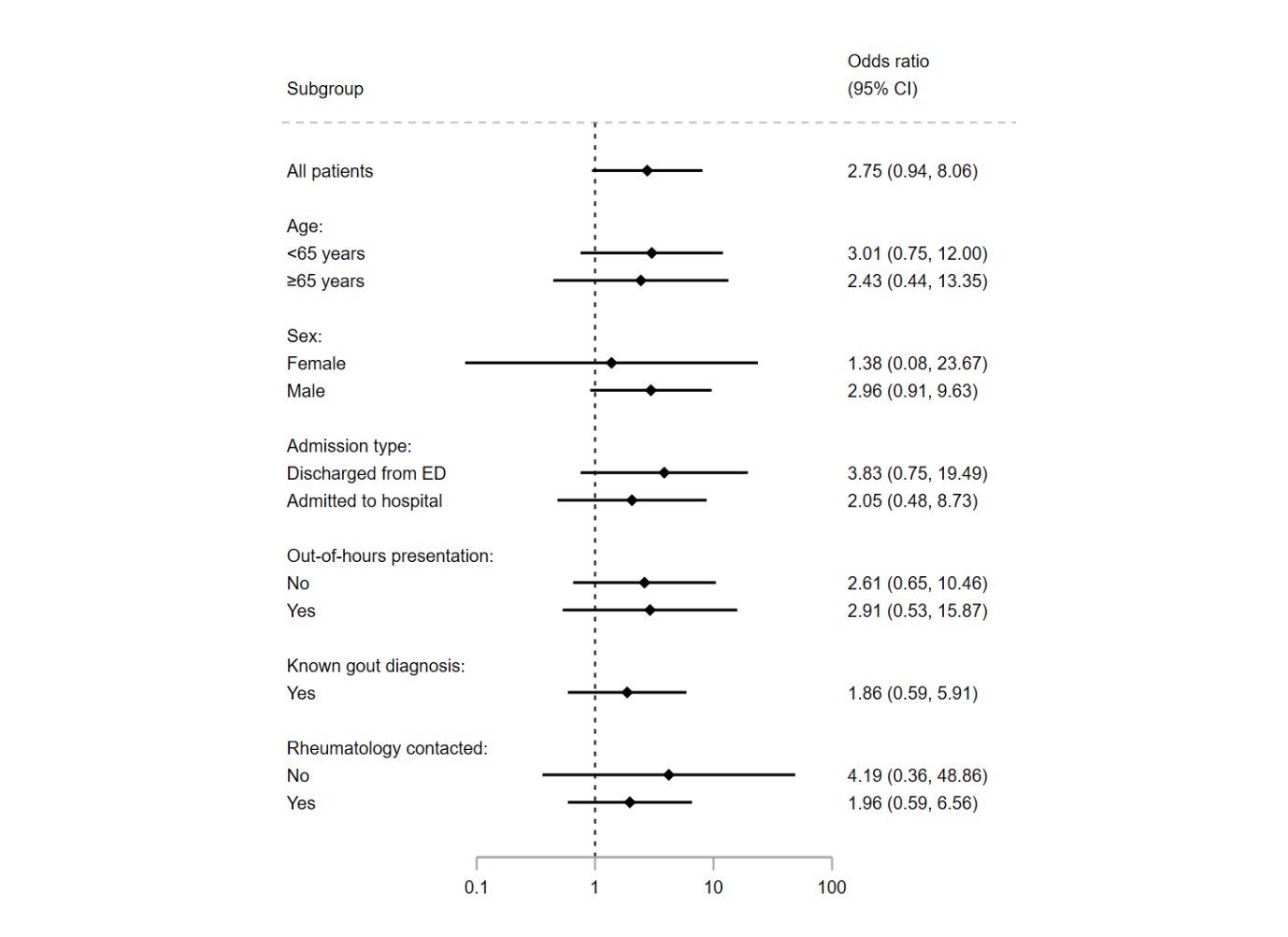
**Supplementary Figure S3.** Odds of achieving a serum urate ≤300 micromol/L within 6 months of discharge in the post-implementation cohort relative to the pre-implementation cohort, comparing different subgroups of patients.

Outputs shown are from univariable logistic regression models.


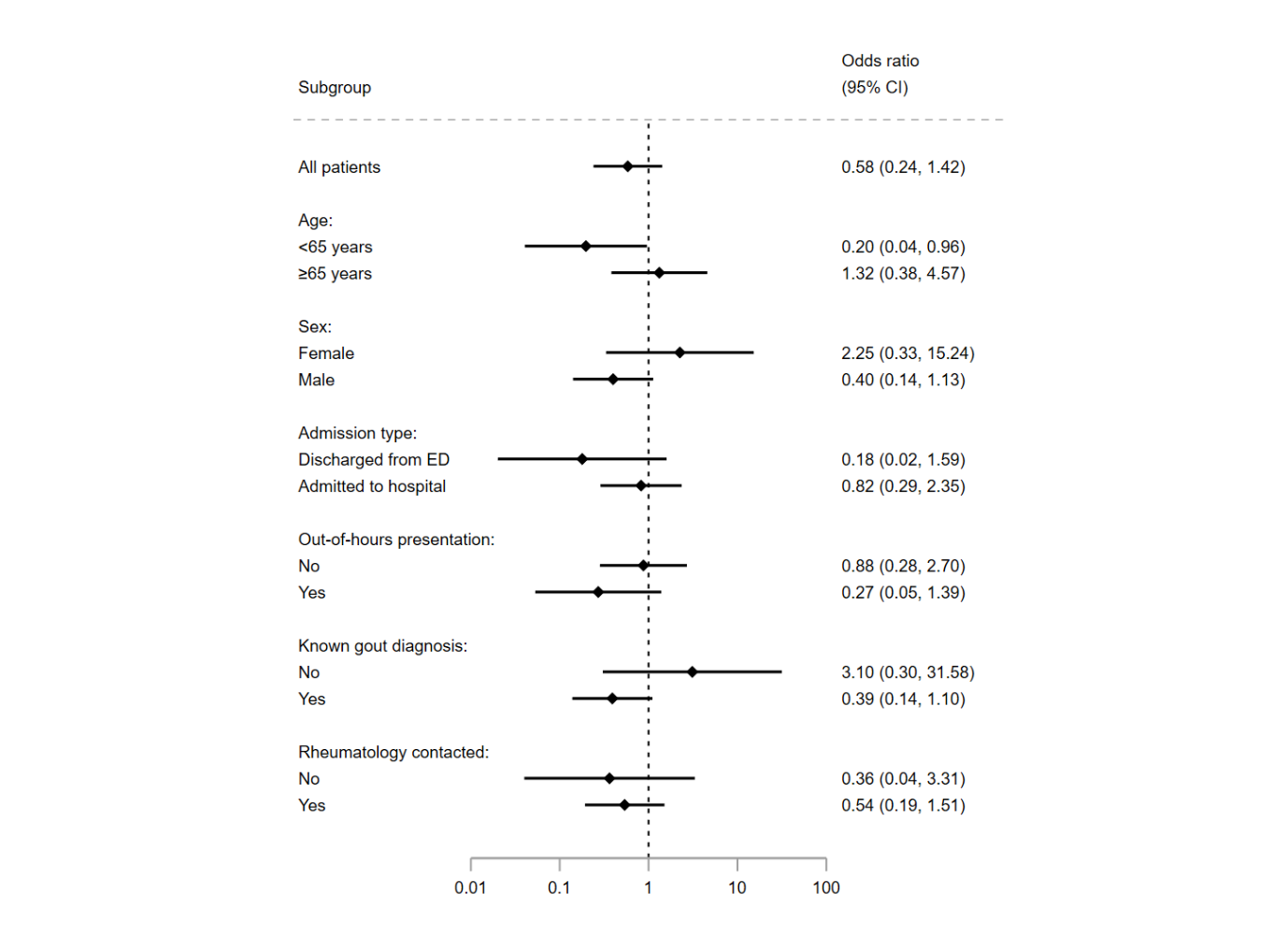
**Supplementary Figure S4.** Odds of re-attending hospital for a gout flare within 6 months of discharge in the post-implementation cohort relative to the pre-implementation cohort, comparing different subgroups of patients.

Outputs shown are from univariable logistic regression models.
